# Safety of Inclisiran: A Disproportionality Analysis from the EudraVigilance Database

**DOI:** 10.3390/ph17101365

**Published:** 2024-10-12

**Authors:** Giuseppe Cicala, Michelangelo Rottura, Viviana Maria Gianguzzo, Federica Cristiano, Selene Francesca Anna Drago, Giovanni Pallio, Natasha Irrera, Egidio Imbalzano, Edoardo Spina, Vincenzo Arcoraci

**Affiliations:** 1 Department of Clinical and Experimental Medicine, University of Messina, 98125 Messina, Italy; gcicala@unime.it (G.C.); cristiano.federica977@gmail.com (F.C.); sedrago@unime.it (S.F.A.D.); nirrera@unime.it (N.I.); eimbalzano@unime.it (E.I.); espina@unime.it (E.S.); varcoraci@unime.it (V.A.); 2 Department of Chemical, Biological, Pharmaceutical and Environmental Sciences, University of Messina, 98166 Messina, Italy; vivianamaria.gianguzzo@unime.it; 3 Department of Biomedical and Dental Sciences and Morphological and Functional Imaging, University of Messina, 98125 Messina, Italy; gpallio@unime.it

**Keywords:** inclisiran, PCSK9 antagonist, alirocumab, evolocumab, adverse drug reactions, pharmacovigilance

## Abstract

**Introduction:** The discovery of serine protease proprotein convertase subtilisin-kexin type 9 (PCSK9) has revolutionized pharmacological lipid-lowering treatments. The first PCSK9 antagonists (PCSK9-A), evolocumab and alirocumab, were approved in 2015. Targeting PCSK9 synthesis marked a major advancement in this field, leading to the development of inclisiran, a long-acting siRNA targeting PCSK9 mRNA. However, real-world safety data on this drug are still limited. Therefore, this study aims to provide a real-world safety evaluation of inclisiran, comparing its characteristics to those of PCSK9-As. **Methods:** A retrospective pharmacovigilance study was conducted using EudraVigilance (EV). Inclisiran-related individual case safety reports (I-ICSRs) from 01/01/2021 to 06/30/2023 were retrieved. ICSRs for evolocumab or alirocumab from 01/01/2015 to 06/30/2023 were collected as a reference group (RG). ADRs were classified using the MedDRA dictionary. Data were evaluated using descriptive and disproportionality analyses. Crude reporting odds ratio (ROR) with 95% confidence intervals (CI) were used as disproportionality measures. **Results:** Of the 15,236 ICSRs, 3.7% (n = 563) involved inclisiran, with the rest in the RG. Most I-ICSRs involved female patients (51.7%) aged 18 to 64 (52.8%). The most-reported ADRs for inclisiran were “general disorders and administration site conditions” (n = 347) and “investigations” (n = 277). Significant disproportionality was found in I-ICSRs compared to the RG for “Myalgia” (ROR: 2.43; 95% CI: 1.94–3.04), “Low-density lipoprotein increased” (ROR: 11.95; 95% CI: 9.10–15.52), and “Drug ineffective” (ROR: 6.37; 95% CI: 4.64–8.74). **Conclusions:** The inclisiran safety profile aligns with the existing literature and pre-commercial data. However, further studies are needed to fully understand the observed differences with PCSK9-As.

## 1. Introduction

The current international cardiovascular (CV) risk management guidelines recommend achieving and maintaining LDL values below strict targets [1,2]. Specifically, the LDL cholesterol target for patients presenting a high/very high CV risk has been lowered to <70/<55 mg/dL (<1.8/<1.44 mmol/L), with a reduction of at least 50% of the baseline value, while for patients who have had two CV events within two years, the guidelines also suggest an LDL cholesterol target of <40 mg/dL (<1 mmol/L) [1,2]. Statins represent the first-line treatment for reaching these goals [1,3]. However, despite the established effectiveness of this class of drugs, the response to statin treatment in clinical practice is known to be highly variable [4]. Data from a systematic review examining studies on patients treated with statins showed that genetic factors play a key role in this context [5]. Furthermore, treatment adherence represents another limiting factor for this therapy, along with the risk of a drug–drug interaction with other drugs that are widely prescribed in general practice [6,7]. Indeed, clinical practice data show that about half of patients treated with statins discontinue the therapy within one year and that more than half of general practitioners co-administer statin and macrolides at high risk of interaction at least once during their daily practice [7,8]. Several pieces of evidence show that, even among compliant patients, only a small portion manages to reach the LDL cholesterol target [9,10]. Moreover, among statin-treated patients at high CV risk and with persistently elevated LDL cholesterol levels, CV event rates remain high [11]. Thus, the development of a new therapeutic strategy for patients resistant to or intolerant of first-line treatment is needed.

The discovery of the role of the serine protease proprotein convertase subtilisin-kexin type 9 (PCSK9) has revolutionized the pharmacological treatment landscape for these patients [12]. In fact, PCSK9 stimulates the degradation of the transmembrane LDL receptor, suggesting an alternative pathway for managing plasma LDL levels [13,14]. A first step towards targeting the PCSK9 regulation pathway was taken with the design of targeted monoclonal antibodies [15]. This approach works by sequestering virtually all PCSK9 in the reticuloendothelial system, thus preventing it from binding to the LDL receptor [16,17]. Evolocumab and alirocumab were the first PCSK9 antagonist (PCSK9-A) drugs approved by the FDA (Food and Drug Administration) and the EMA (European Medicines Agency) in 2015 [18,19]. Several clinical studies regarding the effectiveness and safety of PCSK9-A were conducted [20]. The major issues concerning PCSK9-A are represented by inflammatory reactions at the injection site; muscular symptoms; and effects on carbohydrate metabolism, the levels of fat-soluble vitamins, and neurocognitive processes [21,22,23].

An important advancement in this field has been the targeting of PCSK9 synthesis, which has led to the development of inclisiran, which was approved by the EMA on 9 December 2020 [24]. Inclisiran is a drug based on small interfering RNA (siRNA). Its unique mechanism of action is based on the inhibition of the PCSK9 synthesis within hepatocytes [25]. In detail, inclisiran is a synthetic long-acting siRNA-targeted PCSK9 mRNA conjugated to the triantennary carbohydrate N-acetylgalactosamine (GalNAc). This GalNAc enables precise and targeted drug uptake by hepatocytes [26,27]. Inclisiran specifically binds to the PCSK9 mRNA precursor, preventing its translation and PCSK9 production [13,28]. This makes inclisiran different from previously available PCSK9-A, which acts by exercising an extracellular inhibition [26,27]. Efficacy studies regarding inclisiran highlighted positive results. In particular, an 80% reduction in plasma PCSK9 and a 50% reduction in LDL levels were observed after treatment [29,30]. Furthermore, in a recent meta-analysis, there was a reduction in myocardial infarction risk, but no significant correlations for the occurrence of stroke (whether compound stroke, ischemic stroke, or hemorrhagic stroke), in patients treated with inclisiran were observed [31].

From a safety standpoint, inclisiran was generally safe and well tolerated, showing a placebo-comparable safety profile during phase III studies [29,30,32,33,34,35,36].

This safety profile was confirmed in a recent meta-analysis, in which inclisiran did not increase the overall frequency of adverse events compared to a placebo [31]. However, a higher number of injection-site reactions were reported, none of which were considered serious [31]. In addition to that, post-marketing real-world safety data for inclisiran are not yet available.

Therefore, the aim of this study was to describe the adverse reactions of inclisiran reported and collected in a European database and, secondarily, to compare the safety profile of this new lipid-lowering drug to that of PCSK9-As.

## 2. Results

### 2.1. Descriptive Analysis

Inclisiran-related individual case safety reports (ICSRs) were extracted from the EudraVigilance (EV) database. In addition to that, ICSRs relative to PCSK9-A were also extracted to provide a reference group (RG) for the analyses. A time-based normalization strategy was applied to the ICSRs in the reference group to account for the different times of market presence. The standardized Medical Dictionary for Regulatory Activities (MedDRA) was used to encode suspected adverse drug reactions (ADRs) data. Further details are provided in the methodology section.

The ICSRs reported in the EV database that presented inclisiran or a PCSK-A as a suspected drug were 15,236. Out of these, 3.7% (n = 563) showed inclisiran as a suspect drug. Among the 14,673 RG ICSRs, 9562 (65.2%) had evolocumab as the suspect drug, while 5016 (34.2%) reported alirocumab. Additionally, in 95 (0.6%) cases, both PCSK9as were reported as suspect drugs.

**Figure 1:**
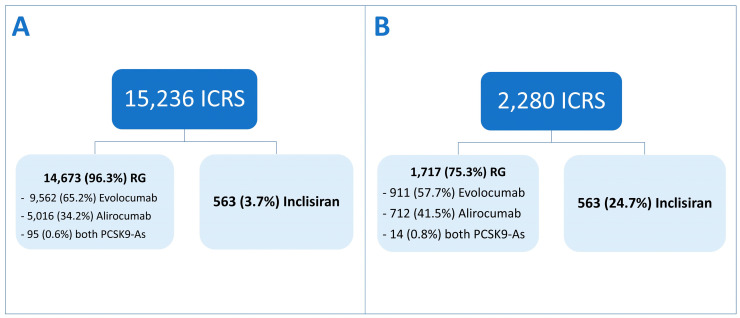
Flowchart study design. (**A**) Non-normalized analysis; (**B**) normalized analysis.

Following normalization, the ICSRs of the RG were 1717, with 991 (57.7%) related to evolocumab, 712 (41.5%) to alirocumab, and 14 (0.8%) presented both drugs (Figure 1).

**Figure 2:**
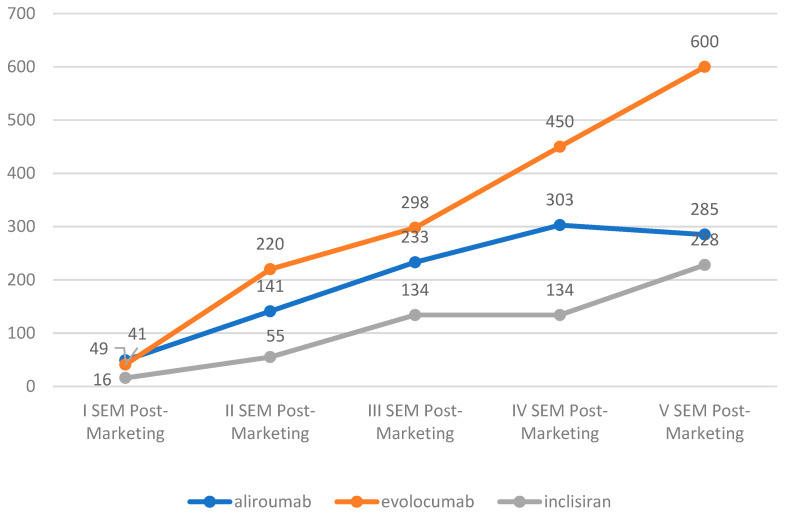
Number of ICSRs of the drugs study in the first 5 semesters post-marketing. SEM, semester.

Both Inclisiran- and PCSK9-As-related ICSRs showed a similarly increasing reporting trend in the first five post-marketing semesters. The only exception was observed for the alirocumab ICSRs, which exhibited a decreasing trend of reports from the fourth to the fifth post-marketing semester. The ICSRs reported for evolocumab were more frequently reported at all time points than for the alirocumab and inclisiran ICSRs (Figure 2).

Most of the inclisiran-related ICSRs did not report patient age (N = 351; 62.3%). The rate of cases with missing age values was lower in the RG for both analysis models.

ICSRs presenting patients with ages <65 years were more common for inclisiran than RG, both in non-normalized and normalized analyses. The frequency of ICSRs with ADRs classified as “serious” was lower in inclisiran ICSRs compared to the RG (non-normalized analysis: 25.0% vs. 58.8%; *p* < 0.001; normalized analysis: 25.0% vs. 81.9%; *p* < 0.001). Additionally, a higher frequency of cases reporting “Fatal” as a possible outcome was observed in the RG compared to inclisiran ICSRs (non-normalized analysis 1.3% vs. 4.4%, *p* = 0.009; normalized analysis 1.3% vs. 6.7%, *p* < 0.001). Conversely, the outcome “Recovered/Resolved With Sequelae” was more frequent in inclisiran ICSRs than in the RG (Non-normalized analysis 1.7% vs. 0.6% and *p* = 0.018; normalized analysis 1.7% vs. 0.2% and *p* = 0.004). A significantly higher frequency of ICSRs reported by the European Economic Area was observed for inclisiran (N = 495; 87.9%) compared to RG (both pre- (N = 7012; 47.7%; *p* < 0.001) and post- (N = 484; 28.2%; *p* < 0.001) normalization). Analyzing only ICSRs from the “European Economic Area” (EEA), the frequency of serious ICSRs in both non-normalized (N = 1301; 18.5%) and normalized (N = 201; 41.5%) RG decreased. Furthermore, considering only EEA ICSRs, no significant differences in serious ICSRs were observed between inclisiran (N = 80; 16.1%) and the RG (N = 1301; 18.5%; *p* = 0.205). However, also in this case, after normalizing the RG subgroup for the time of market presence, a significantly lower frequency of serious ICSRs was observed for inclisiran compared to RG (N = 201; 41.5%; *p* < 0.001). The main characteristics of ICSRs related to inclisiran compared to PCSK9a are summarized in Table 1.

**Figure 3:**
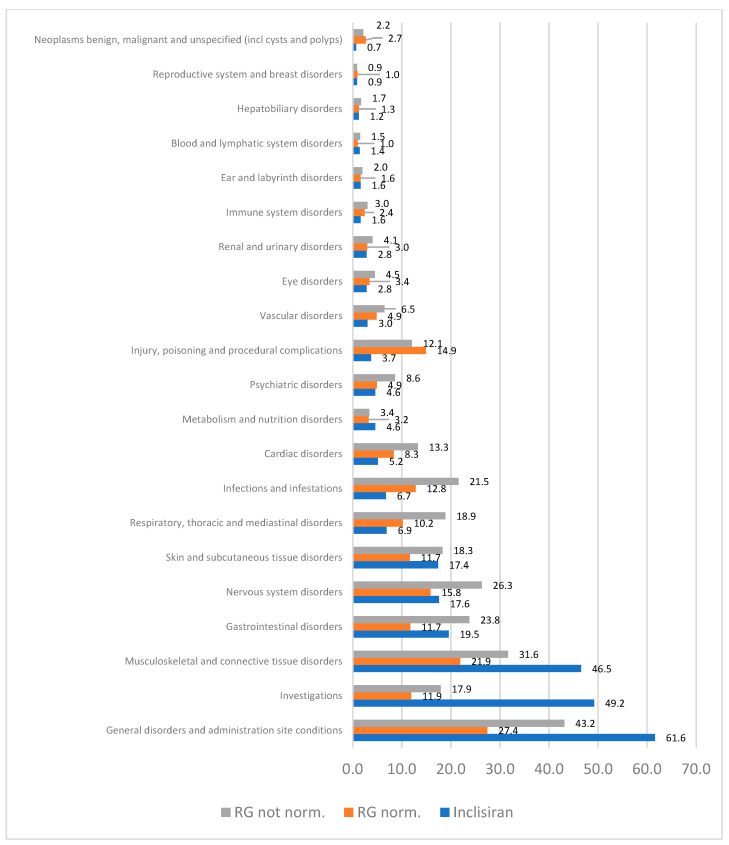
MedDRA^®^ system organ class according to study group.

A total of 37,680 suspected adverse reactions were observed in the retrieved ICSRs, accounting for a mean of 2.5 reactions per ICSR. In particular, 1468 suspected reactions were reported in the inclisiran ICSRs, with a mean of 2.6 reactions per ICSR, while 36,212 suspected reactions were reported in the RG ICSRs, with a mean of 2.4 reactions per ICSR. After stratifying the observed ADRs in the MedDRA^®^ system organ classes (SOC), the most reported SOCs in the inclisiran ICSRs were “general disorders and administration site conditions” (N = 347; 61.6%), “investigations” (N = 277; 49.2%), and “musculoskeletal and connective tissue disorders” (N = 262; 46.5%). The SOCs observed to have a higher frequency in inclisiran compared to normalized and non-normalized RG were “general disorders and administration site conditions”, “investigations”, “musculoskeletal and connective tissue disorders”, and “metabolism and nutrition disorders”. Conversely, the SOCs less frequently reported in the inclisiran ICSRs compared to the RG were “neoplasms benign, malignant and unspecified (incl cysts and polyps)”, “immune system disorders”, “renal and urinary disorders”, “eye disorders”, “vascular disorders”, “injury, poisoning and procedural complications”, “psychiatric disorders”, “cardiac disorders”, “infections and infestations”, and “Respiratory, thoracic and mediastinal disorders” (Figure 3).

### 2.2. Disproportionality Analysis

Disproportionately reported ADRs in the inclisiran ICSRs were evaluated in comparison to those observed in the PCSK9-As. The chosen disproportionality measure was the reporting odds ratio with associated 95% confidence intervals (CI). Further details are provided in the methods section.

For all three of the most frequently reported MedDRA^®^ preferred terms (PTs) in the inclisiran ICSRs, significant disproportionalities were observed compared to the RG. These results were confirmed in both non-normalized and normalized analyses; “myalgia”, (ROR [95%CI]: normalized analysis = 2.43 [1.83, 3.10]; non-normalized analysis = 2.43 [1.94, 3.04], “low density lipoprotein increased”, (ROR [95%CI]: normalized analysis = 13.25 [8.34, 21.03]; non-normalized analysis = 11.95 [9.10, 15.52], and “drug ineffective” (ROR [95%CI]: normalized analysis = 6.74 [4.14, 10.99]; non-normalized analysis = 6.37 [4.64, 8.74]. Among those, only the ADRs “Injection site erythema”, “injection site pain”, “injection site reaction”, and “injection site rash” were reported in the inclisiran summary of product characteristics (SmPC). See Table 2 and Table S1 for further details.

In addition, the PTs present in the RG group that were not reported in the inclisiran group, such as “myocardial infarction”, “pneumonia”, and “angina pectoris”, were also observed. See Tables S2 and S3 for further details.

## 3. Discussion

### 3.1. Descriptive Analysis

In this study, we evaluated the safety profile of inclisiran using the ICSR data from the EV database and compared it to those related to PCSK9-As. Inclisiran-related ICSRs were in lower numbers for each post-marketing semester than the PCSK9-As ICSRs. Evolocumab was the PCSK9-A with the highest number of associated ICSRs. This is in line with other pharmacovigilance studies [37,38]. An upward trend in ICSR reporting frequency for both inclisiran and PCSK9-A ICSRs was observed. This trend can be explained by the interest these drugs are receiving from specialists. Indeed, after their marketing authorization, the use of both inclisiran and PCSK9-As has shown a constant increase [39,40,41]. However, the influence of the so-called “Weber effect” cannot be excluded. This name identifies the phenomenon for which the yearly reporting frequency of ICSRs is higher in the first marketing period of a drug [38]. A consequence of this effect is that most ICSRs reported in this first phase usually concern non-serious ADRs. This is because ADRs associated with new drugs tend to attract more attention. Meanwhile, for already established drugs, more attention is given to serious ADRs, thus resulting in lower reporting of non-serious ADRs. Surprisingly, a shift in the serious-to-non-serious ratio can be observed in our analysis by considering the RG data. Indeed, after the period considered in normalized RG, a substantial increase in the percentage of “non-serious” ICSRs was observed. However, the date of marketing of the drugs must be considered. Indeed, pharmacovigilance has undergone significant evolution in recent decades. Both the spontaneous reporting systems of suspected ADRs and the sensitivity of healthcare professionals to report any ADR, even those not considered serious, have improved [42]. Both evolocumab and alirocumab received marketing authorization in 2015, approximately 10 years before inclisiran [18,19,24]. Therefore, the high frequency of serious ADRs in normalized RG could be attributed to the marketing period of these drugs. Furthermore, our data revealed a significantly higher reporting of severe ICSRs in RG compared to inclisiran. However, this data could be highly distorted by the ICSRs of the “Non-European Economic Area”. In fact, from the comparative analysis of the ICSRs of the “European Economic Area” alone, although in inclisiran the prevalence of serious ICSRs remains lower (16.1%) compared to RG (18.5%), no significant differences were observed. Therefore, only limited conclusions can be drawn regarding the seriousness of ADRs related to inclisiran compared to those related to PCSK9-As. In our opinion, the implementation of international guidelines would be appropriate to conform to the ADRs’ seriousness evaluation methodology between different geographical areas. Such standardization of data would facilitate the comparison of information relating to different countries. Patient characteristics exhibited differences between inclisiran-related ICSRs and those related to the RG. A higher frequency of reports involving patients under 65 years old was noticed for inclisiran, both before and after the time normalization of the RG. This aligns with the real-world context of these data. It is, in fact, usual to prefer a more consolidated therapeutic option to treat elderly patients. This difference persisted even when considering the time-normalized RG. However, at the time of PCSK9-As commercialization, no other therapeutic strategies with a similar safety profile were available. Therefore, the use of PCSK9-As in elderly patients was reasonably more frequent [39,40,43]. The SOC observed with the highest frequency for inclisiran was “general disorders and administration site conditions”. This is in line with the limited safety data available at the moment in the literature for this drug. In the ORION-3 study (extension study of the phase-2 ORION-1 trial), 27.8% of the enrolled subjects had at least one adverse event possibly related to inclisiran. The most frequently reported ADRs in the trial were “injection site reactions” (5.6%), “injection site erythema” (4.2%), “injection site pain” (4.2%), “injection site rash” (2.8%), “myalgia” (2.5%), “injection site bruising” (1.4%), “hepatic enzyme increased” (1.4%), “muscle spasms” (1.4%), “fatigue” (1.1%), “injection site discoloration” (1.1%), and “pain in extremity” (1.1%) [44]. All adverse events reported in the clinical trials were observed as ADRs in our study.

### 3.2. ADRs of Clinical Interest

#### 3.2.1. Myalgia Related ADRs

The most frequently reported PT for both inclisiran and RG was “myalgia” (inclisiran: 17.5%; RG normalized analysis: 8.2%; RG non-normalized analysis: 8.0%). Disproportionate odds of reporting were observed for this PT both before and after normalization of the RG. Also, the PT “pain in extremity” was found as disproportional. Considering the literature sources, “Pain in extremity” and “Myalgia” were reported as adverse events in 8.8% and 8.5% of participants in an inclisiran clinical trial, respectively [44]. In accordance with this, data from a recent real-world study conducted on the FDA Adverse Event Reporting System (FAERS) suggested myalgia as a possible safety signal related to inclisiran [45]. This ADR was also reported as one of the most frequently observed in relation to PCSK9-A in a previous pharmacovigilance study [46]. Moreover, a study conducted using clinical practice data highlighted how 15.0% of inclisiran-treated patients reported “Muscle pain” [47]. However, the results of a meta-analysis that evaluated the efficacy and safety profile of the two PCSK9-As did not associate an increased risk of myalgia among users of these drugs [48]. A recent study utilizing network meta-analysis (NMA) suggests that lipid-lowering therapies, including those prescribed alongside statins, could impact the musculoskeletal system. It has been observed that adverse drug reactions are both more severe and more frequent in female subjects compared to males. This underscores the need for further studies specifically focusing on inclisiran [49]. However, the observation of these ADRs, both in relation to inclusion and PCSK9-As, might hint at an underlying mechanism that is dependent upon the PCSK9 levels rather than the administration of the drugs. Given the current uncertainty about the underlying mechanism and the relative novelty of inclisiran, in our opinion, the onset of these muscle-related adverse events requires careful monitoring.

#### 3.2.2. Drug Ineffectiveness

ADRs related to insufficient lipid control such as “Low density lipoprotein increased” (15.8%) and “drug ineffective” (9.0%) represented the second and third most frequently reported PTs in inclisiran ICSRs. In addition, a significantly higher probability of reporting was observed in the inclisiran ICSRs compared to those of PCSK9-As for PTs related to treatment failure, such as “Low density lipoprotein increased”, “Drug ineffective”, “Blood cholesterol increased”, and “Drug effect less than expected”. A high variability in cholesterol changes was observed in the clinical trials and post-marketing observational studies regarding inclisiran use [44,47]. However, LDL levels increased in patients who switched from PCSK9-A to inclisiran and never in patients starting lipid-lowering therapy [44,47]. Furthermore, the influence of selection bias in our study cannot be ruled out. A recent observational study showed that patients treated with inclisiran who previously received therapy with PCSK9-As experienced fewer effective reductions in LDL cholesterol [50]. Indeed, patients treated with PCSK9-As are generally characterized by higher baseline LDL levels compared to those treated with other lipid-lowering drugs [51,52]. Thus, this might represent a predisposing factor for an ineffective inclisiran treatment. In our opinion, further prospective studies with drug-naïve populations might be necessary to properly assess these aspects.

#### 3.2.3. Weight Gain

The disproportionality analyses showed a greater probability of reporting “Weight gain” in inclisiran ICSRs compared to PCSK9-A ones. This observation seems to contradict the established pathophysiological mechanisms associated with PCSK9 [53]. Furthermore, two studies observed a direct association between the increase in PCSK9 levels and obesity [54,55]. Thus, in our opinion, these data might be influenced by the presence of an indication bias regarding these ADRs. Moreover, this ADR was reported in a limited number of ICSRs (n = 13; 2.3%). After an in-depth evaluation of these cases, we observed that the mean number of ADRs per ICSR was 4.6 (±2.4 SD). This indicates that most of these ICSRs described complex conditions. Unfortunately, the lack of patient medical histories limits our possibility of drawing conclusions regarding these manifestations.

#### 3.2.4. Injection-Site Reactions

PTs related to injection-site conditions (“injection site erythema”, “injection site pain”, “injection site pruritus”, “injection site reaction”, and “injection site rash”) were reported overall in 14.5% of inclisiran ICSRs. Furthermore, the disproportionality analysis highlighted a significantly greater probability of reporting ADRs related to the injection site (Table S1) in inclisiran ICSRs compared to RG, both in the non-normalized and normalized analyses. With the exception of “injection site pruritus”, these ADRs are already acknowledged in the inclisiran SmPC [24]. From a literature perspective, in a recent systematic review, a higher incidence of injection-site reactions was observed in the inclisiran-treated patients compared to placebo-treated ones (RR = 6.2, 95%CI = 2.6, 14.9) [56]. However, the recent literature data have highlighted that injection-site reactions seem to be a class effect of all SiRNA-based therapies rather than inclisiran-specific ADRs [57]. Even if in-depth pharmacodynamic evaluation exceeds the scope of our study, these data, in our opinion, need further investigation.

#### 3.2.5. Changes in Liver Functions

In our study, 3.5% of the inclisiran ICSRs reported an ADR related to changes in liver function parameters (liver function test increased and transaminases increased). Furthermore, an increased probability of reporting ADRs related to liver dysfunction emerged in inclisiran ICSRs compared to RG ones, both in the normalized and non-normalized analysis. ADRs related to liver dysfunction have already been reported in the Orion 3 clinical trial. In particular, 3% of patients treated with inclisiran after a therapeutic switch from PCSK9-A presented increases in liver enzymes believed to be related to the inclisiran. In fact, PCSK9-A presents a generally good hepatic safety profile, as demonstrated by a recent meta-analysis [58]. Therefore, possible ADRs involving the hepatic dysfunction observed with inclisiran could be attributed to its innovative formulation and hepatic selectivity. In our opinion, careful liver function monitoring should be maintained in patients treated with inclisiran. In addition to that, molecular studies should be undertaken to investigate this condition further.

#### 3.2.6. Strengths and Limitations

This is the first European study to focus on ADRs related to the use of inclisiran compared to those of evolocumab and alirocumab using a spontaneous reporting system (SRS) database. Through the use of a large-scale SRS database, we were able to obtain real-world insights into the safety of inclisiran. However, pharmacovigilance studies have some inherent limitations, including an under-reporting of suspected ADRs. In fact, SRSs are mainly based on the motivation of individuals to report suspected ADRs to a local or national pharmacovigilance center [59]. Additionally, a significant limitation is the lack of data on the denominator (i.e., the total number of inclisiran users). This prevents calculating the absolute risk of suspected ADRs. This also represents an issue common to all studies using SRSs’ collected data. While these studies are valuable, like in our case, for generating safety hypotheses [60], the absence of denominator data highlights the need for further post-marketing surveillance trials to obtain comparative ADR incidence rates [61,62]. Moreover, the publicly accessible version of EV has some specific limitations, such as the absence of data regarding the entirety of the database. This limited our capabilities in terms of global safety evaluations. However, the choice of a reference group constituted by PCSK9-A ICSRs allowed us to observe safety differences between these two treatment approaches, which might be of interest to clinicians. The two different commercialization periods of inclisiran and PCSK9-As could have introduced biases, such as the Webber effect, that might have influenced our evaluations. To minimize these biases, we introduced a time-based normalization strategy that considered an analogous time frame for both inclisiran and PSCK9-As. Furthermore, the MedDRA classification has some limitations, particularly in identifying the correct PT, which may be reported with different PT synonyms for the same clinical condition, leading to misrepresentation of reported ADRs [63].

## 4. Materials and Methods

### 4.1. Data Source and Selection Process

A retrospective pharmacovigilance study was conducted using the EV platform (publicly available at www.adrreports.eu, accessed on 15 July 2023). EV serves as the final collection point for the ADRs Spontaneous Reporting Systems of the European Union. The database was created to facilitate the management and analysis of information on suspected ADRs. EV includes both data relative to medications in their post-marketing phase and on drugs currently under evaluation in clinical trials. Several studies have already highlighted the importance of the EV as a tool for obtaining real-world safety data [64,65].

### 4.2. Reports Selection

Data on ICSRs presenting inclisiran as a suspected drug were retrieved from the EV database. The timeframe considered for inclisiran-related ICSRs was between 1 January 2021 (the year of inclisiran first market approval) and 30 June 2023. ICSRs presenting as suspected drugs the previously available PCSK9-A (alirocumab and evolocumab) were also retrieved to constitute the RG for the analyses. The time interval considered for RG cases was between 1 January 2015 (the year of commercialization of both alirocumab and evolocumab) and 30 June 2023. Cases linked to the literature sources or presenting vaccines as suspected drugs were excluded.

### 4.3. Data Characteristics

The retrieved ICSRs included information regarding their EV case identification number, the report compilation date, and the primary source qualification. The extracted ICSRs also included information regarding patient gender, age group, ADR characteristics (type of ADR, duration, outcome, and seriousness criteria), and characteristics of suspected and concomitant drugs (type of drug, indication of use, duration of therapy, drug dose, and administration route).

All ADRs were classified in accordance with MedDRA^®^ version 26.0. MedDRA is structured hierarchically, with terms organized into five levels from the most specific to the broadest, including “Lowest-Level Terms”, “Preferred Term”, High-Level Terms”, “High-Level Group Terms”, and “System Organ Classes”. The MedDRA^®^ structure has been described in detail in previous studies [66]. The ADRs were classified as “serious” when linked to the death or life-threatening of the patient, if caused or prolonged hospitalization, persistent disability/incapacity, a congenital anomaly/birth defect, or conditions deemed as medically important by the reporter. For the ADR outcomes, standardized terminology was used, with ADRs classified as: “recovered/resolved”, “recovering/resolving”, “recovered/resolved with sequelae”, “not recovered/not resolved”, “fatal”, and “unknown” on the bases of what was reported in the ICSR. All drugs were classified according to the Anatomical Therapeutic and Chemical (ATC) Classification System. The ADRs’ expectedness was verified based on the summary of product characteristics (SmPCs) available in the EMA database. If different outcomes due to two or more ADRs were reported in the same ICSR, a global outcome for the case described in the ICSR was estimated using the “Lower Level of Resolution” methodology, as previously described [64,67].

### 4.4. Data Analysis

The demographic characteristics of the patients, characteristics of the reports, and adverse events were evaluated with a descriptive analysis. Categorical variables were summarized as absolute frequencies and percentages. Two-tailed Pearson chi-square tests were adopted to compare the previously stated characteristics between the ICSR groups. Values of *p* < 0.05 were considered statistically significant.

A disproportionality analysis was performed using the RORs, with the corresponding 95% CI, to assess the possible differences in the reporting odds of ADRs. The analysis was conducted at the MedDRA^®^ SOC and the PT Term level. The statistical significance threshold was defined as a lower 95% CI limit of >1 in cases of a positive association, while there was an upper 95% CI limit of <1 in cases of a negative association. Disproportionality analyses were performed only for the ADRs reported in at least three ICSRs. Additionally, in order to normalize the analysis to the same observation period starting from commercialization for both study groups, a subanalysis (normalized analysis) was conducted, including ICSRs from the RG reported only between 1 January 2015, and 30 June 2017 (the first 2 years and 6 months after the commercialization of the PCSK9-A), as well as for inclisiran. Disproportional ADRs were further investigated only if the ROR was still significant after the normalization of the RG. All analyses were carried out using SPSS (Statistical Package for the Social Sciences, IBM Corp., Armonk, NY, USA) Version 28. In addition to that, ADR expectedness was evaluated based on the information available in the inclisiran SmPC released by the European Medicines Agency.

## 5. Conclusions

We carried out a descriptive and statistical analysis of data from 563 ICSRs related to inclisiran and 14,697 related to evolocumab and alirocumab. Overall, the safety profile of inclisiran was found to be coherent with what is currently known from the literature data and randomized controlled studies. We found that inclisiran had disproportional reporting odds in relation to muscle pain, therapeutic ineffectiveness, injection-site reactions, and liver dysfunction compared to PCSK9-As. However, no cardiovascular side effects were reported. Our findings could be a useful tool to assist cardiologists in managing ADRs in clinical practice. Further analyses should be conducted to better contextualize some of the safety aspects of inclisiran, such as injection-site reactions and changes in liver functions. The in-depth evaluation of these ADRs could allow for a better characterization of the inclisiran safety profile compared to PCSK9-As, which is of crucial importance considering their current interchangeability of use in clinical practice. Furthermore, the results of our analysis highlight how close collaboration between cardiologists and pharmacologists could improve their management capabilities, improve ICSR reporting, and increase the real-world knowledge of prescribed drug therapies.

## Figures and Tables

**Table 1:** Characteristics of the ICSRs related to inclisiran compared to those related to other iPCSK9 drugs.

Characteristics	Inclisiran ICSRs N 563 (%)	RG Norm N (%)	*p*-Value Inclisiran vs. RG Norm	RG No-Norm N (%)	*p*-Value Inclisiran vs. RG No-Norm
Sex					
Male	264 (48.3)	779 (47.2)	0.653	6475 (46.1)	0.313
Female	283 (51.7)	873 (52.8)	0.653	7579 (53.9)	0.313
Age categories (years)					
<65	112 (52.8)	520 (42.9)	**0.014**	3877 (42.8)	**0.003**
≥65	100 (47.2)	670 (57.1)	0.014	5204 (57.2)	**0.003**
Unknown	351 (62.3)	505 (29.4)	-	5599 (38.1)	-
Seriousness					
Serious	141 (25.0)	1406 (81.9)	**<0.001**	8634 (58.8)	**<0.001**
Non-serious	422 (75.0)	311 (18.1)	**<0.001**	6039 (41.2)	**<0.001**
Outcome					
Fatal	4 (1.3)	62 (6.7)	**<0.001**	305 (4.4)	**0.009**
Not Recovered/Not Resolved	123 (40.6)	320 (34.7)	0.064	2522 (36.7)	0.171
Recovered with Sequelae	5 (1.7)	2 (0.2)	**0.004**	39 (0.6)	**0.018**
Recovering/Resolving	55 (18.2)	135 (14.6)	0.143	1165 (16.9)	0.585
Recovered/Resolved	116 (38.3)	403 (43.7)	0.097	2838 (41.3)	0.293
Unknown	260 (46.2)	795 (46.3)	-	3677 (25.1)	-
Primary Source Qualification					
Healthcare Professional	486 (86.3)	1336 (77.8)	**<0.001**	10967 (74.7)	**<0.001**
Non-Healthcare Professional	77 (13.7)	381 (22.2)	**<0.001**	3706 (25.3)	**<0.001**
Country					
European Economic Area	495 (87.9)	484 (28.2)	**<0.001**	7012 (47.7)	**<0.001**
Non-European Economic Area	68 (12.1)	1233 (71.8)	**<0.001**	7685 (52.3)	**<0.001**

Reference group = RG. Significant values of *p* are reported in bold.

**Table 2:** Disproportionalities in inclisiran ICSRs at the MedDRA PT level.

Preferred Terms	N	Inclisiran vs. RG Norm. ROR [95%CI]	Inclisiran vs. RG No-Norm. ROR [95%CI]	Exp.
Drug intolerance	13	13.50 [3.83, 47.56]	5.30 [2.95, 9.85]	No
Low-density lipoprotein increased	89	13.25 [8.34, 21.03]	11.95 [9.10, 15.52]	No
Injection-site erythema	35	8.06 [4.31, 15.10]	5.19 [3.58, 7.53]	Yes
Drug ineffective	51	6.74 [4.14, 10.99]	6.37 [4.64, 8.74]	No
Liver function test increased	13	5.05 [2.08, 12.25]	13.85 [7.05, 27.22]	No
Injection-site pruritus	17	4.83 [2.25, 10.37]	4.01 [2.39, 6.73]	No
Injection-site reaction	15	4.25 [1.94, 9.20]	5.71 [3.25, 10.04]	Yes
Weight increased	13	4.03 [1.76, 9.25]	2.40 [1.35, 4.26]	No
Injection-site pain	27	3.88 [2.19, 6.87]	1.55 [1.04, 2.30]	Yes
Blood cholesterol increased	19	3.71 [1.89, 7.27]	3.94 [2.41, 6.42]	No
Drug effect less than expected	8	3.52 [1.27, 9.75]	3.14 [1.50, 6.57]	No
Transaminases increased	7	3.59 [1.20, 10.73]	4.98 [2.21, 11.22]	No
Inappropriate schedule of product administration	7	3.59 [1.20, 10.73]	2.66 [1.22, 5.83]	No
Blood triglycerides increased	13	3.36 [1.52, 7.40]	5.00 [2.75, 9.10]	No
Myalgia	99	2.42 [1.83, 3.10]	2.43 [1.94, 3.04]	No
Pain in extremity	24	1.69 [1.02, 2.81]	1.55 [1.02, 2.36]	No

Table sorted by decreasing reporting odds ratio values; no-norm., no-normalized analysis; norm., normalized analysis; PT = preferred term; RG, reference group; ROR, reporting odds ratio.

## Data Availability

This study was entirely based on publicly anonymized data made available by the European Medicines Agency. A preprocessed version of the data can be downloaded at the following link: https://www.adrreports.eu/ (accessed on 15 July 2023).

## Supplementary Materials

The following supporting information can be downloaded at https://www.mdpi.com/article/10.3390/ph17101365/s1: Table S1: Disproportionalities in inclisiran-related ICSRS compared to RG (normalized and non-normalized analysis) stratified by PT; Table S2: The 20 most frequent PTs observed in RG in the non-normalized analysis not reported in inclisiran; Table S3: The 20 most frequent PTs observed in RG in the normalized analysis not reported in inclisiran.
