# Supplementary material for: Safety of Inclisiran: A Disproportionality Analysis from the EudraVigilance Database
Source: Pharmaceuticals (Basel). 2024 Oct 12;17(10):1365. doi: 10.3390/ph17101365 (PMC11511047; doi:10.3390/ph17101365)
Supplement: Supplementary file 1 [file pharmaceuticals-17-01365-s001.zip › pharmaceuticals-3240857-supplementary.pdf]

Supplementary Material Table S1. Disproportionalities in inclisiran-related ICSRS compared to RG (normalized and non-normalized analysis) stratified by PT.

MedDRA PT	Inclisiran N	Inclisiran vs RG norm. ROR [95%CI]	Inclisiran vs RG no-norm. ROR [95%CI]	Exp.
Drug intolerance	13	13.5 [3.8-47.5]	5.3 [2.9-9.8]	NO
Low density lipoprotein increased	89	13.2 [8.3-21.0]	11.9 [9.1-15.5]	NO
Injection site erythema	35	8.0 [4.3-15.1]	5.1 [3.5-7.5]	YES
Drug ineffective	51	6.7 [4.1-10.9]	6.3 [4.6-8.7]	NO
Liver function test increased	13	5.0 [2.0-12.2]	13.8 [7.0-27.2]	NO
Injection site pruritus	17	4.8 [2.2-10.3]	4.0 [2.3-6.7]	NO
Injection site reaction	15	4.2 [1.9-9.2]	5.7 [3.2-10.0]	YES
Weight increased	13	4.0 [1.7-9.2]	2.4 [1.3-4.2]	NO
Injection site pain	27	3.8 [2.1-6.8]	1.5 [1.0-2.3]	YES
Blood cholesterol increased	19	3.7 [1.8-7.2]	3.9 [2.4-6.4]	NO
Drug effect less than expected	8	3.5 [1.2-9.7]	3.1 [1.5-6.5]	NO
Transaminases increased	7	3.5 [1.2-10.7]	4.9 [2.2-11.2]	NO
Inappropriate schedule of product administration	7	3.5 [1.2-10.7]	2.6 [1.2-5.8]	NO
Blood triglycerides increased	13	3.3 [1.5-7.4]	5.0 [2.7-9.1]	NO
Myalgia	99	2.4 [1.8-3.1]	2.4 [1.9-3.0]	NO
Pain in extremity	24	1.6 [1.0-2.8]	1.5 [1.0-2.3]	NO
Therapeutic product effect incomplete	5	5.1 [1.2-21.4]	1.7 [0.7-4.3]	NO
Gamma-glutamyltransferase increased	4	4.0 [0.9-18.3]	4.1 [1.4-12.0]	NO
Myopathy	4	4.0 [0.9-18.3]	3.3 [1.1-9.6]	NO
Therapeutic product effect decreased	4	4.0 [0.9-18.3]	1.2 [0.4-3.4]	NO
Bone pain	5	3.8 [1.0-14.3]	2.2 [0.9-5.6]	NO
Joint swelling	6	3.0 [0.9-9.5]	3.2 [1.3-7.7]	NO
Arrhythmia	5	3.0 [0.8-10.6]	3.8 [1.5-9.9]	NO
C-reactive protein increased	4	3.0 [0.7-12.2]	5.5 [1.8-16.2]	NO
Restlessness	4	3.0 [0.7-12.2]	2.8 [1.0-7.9]	NO
Limb discomfort	3	3.0 [0.6-15.2]	1.3 [0.4-4.3]	NO
Injection site rash	6	2.6 [0.8-7.8]	2.8 [1.2-6.5]	YES
Low density lipoprotein decreased	9	2.5 [1.0-6.1]	3.4 [1.7-6.9]	NO
Muscle disorder	4	2.4 [0.6-9.1]	2.5 [0.9-7.1]	NO
Alopecia	4	2.4 [0.6-9.1]	1.0 [0.3-2.7]	NO
Product use in unapproved indication	7	2.3 [0.8-6.4]	1.1 [0.5-2.4]	NO
Inflammation	3	2.2 [0.5-10.2]	2.8 [0.8-9.2]	NO
Loss of consciousness	6	2.0 [0.7-5.7]	2.3 [1.0-5.4]	NO
Dyspepsia	4	2.0 [0.5-7.2]	2.6 [0.9-7.5]	NO
Injection site discolouration	3	1.8 [0.4-7.6]	3.9 [1.1-13.2]	NO
Erythema	11	1.6 [0.8-3.5]	1.7 [0.9-3.2]	NO
General physical health deterioration	4	1.5 [0.4-5.0]	2.9 [1.0-8.4]	NO
Syncope	4	1.5 [0.4-5.0]	2.3 [0.8-6.4]	NO
Rash pruritic	3	1.5 [0.3-6.1]	0.8 [0.2-2.8]	NO
Hepatic enzyme increased	6	1.4 [0.5-3.7]	2.9 [1.2-6.8]	NO
Arthralgia	42	1.3 [0.9-1.9]	1.2 [0.8-1.6]	NO
Muscle spasms	20	1.3 [0.7-2.2]	1.5 [0.9-2.4]	NO
Transient ischaemic attack	4	1.3 [0.4-4.4]	1.8 [0.6-5.1]	NO
Hypoaesthesia	5	1.3 [0.4-4.0]	1.5 [0.6-3.8]	NO

Dysgeusia	3	1.3 [0.3-5.0]	2.0 [0.6-6.5]	NO
Diarrhoea	21	1.2 [0.7-2.0]	1.6 [1.0-2.6]	NO
Peripheral swelling	5	1.2 [0.4-3.6]	1.1 [0.4-2.9]	NO
Malaise	12	1.1 [0.6-2.3]	1.1 [0.6-2.0]	NO
Pruritus	21	1.1 [0.6-1.8]	1.2 [0.8-2.0]	NO
Blood pressure increased	9	1.1 [0.5-2.6]	1.6 [0.8-3.2]	NO
Blood creatine phosphokinase increased	9	1.1 [0.5-2.6]	1.5 [0.7-3.0]	NO
Muscular weakness	9	1.1 [0.5-2.4]	1.3 [0.6-2.6]	NO
Gait disturbance	7	1.1 [0.4-2.8]	1.2 [0.5-2.6]	NO
Abdominal discomfort	7	1.1 [0.4-2.6]	2.0 [0.9-4.3]	NO
Flatulence	3	1.1 [0.3-4.3]	2.5 [0.7-8.3]	NO
Anaphylactic reaction	3	1.1 [0.3-4.3]	2.0 [0.6-6.7]	NO
Arthropathy	4	1.1 [0.3-3.4]	1.9 [0.7-5.4]	NO
Palpitations	4	1.1 [0.3-3.4]	1.1 [0.4-3.2]	NO
Visual impairment	4	1.1 [0.3-3.4]	1.1 [0.4-3.2]	NO
Pain	19	1.0 [0.6-1.7]	1.5 [0.9-2.4]	NO
Headache	22	1.0 [0.6-1.7]	1.2 [0.7-1.8]	NO
Fatigue	29	1.0 [0.6-1.6]	1.4 [1.0-2.2]	NO
Depression	4	1.0 [0.3-3.1]	1.2 [0.4-3.3]	NO
Weight decreased	5	1.0 [0.3-2.8]	0.9 [0.3-2.2]	NO
Blood creatinine increased	3	1.0 [0.2-3.7]	2.3 [0.7-7.5]	NO
Injection site swelling	3	1.0 [0.2-3.7]	0.5 [0.1-1.6]	NO
Back pain	14	0.9 [0.4-1.6]	0.8 [0.5-1.5]	NO
Sleep disorder	4	0.9 [0.3-2.8]	1.5 [0.5-4.2]	NO
Pyrexia	10	0.8 [0.4-1.7]	1.4 [0.7-2.7]	NO
Acute myocardial infarction	3	0.8 [0.2-2.9]	1.3 [0.4-4.4]	NO
Alanine aminotransferase increased	3	0.8 [0.2-2.9]	1.3 [0.4-4.3]	NO
Constipation	4	0.8 [0.2-2.4]	1.3 [0.5-3.7]	NO
Rash	18	0.7 [0.4-1.2]	1.1 [0.6-1.8]	NO
Nausea	18	0.7 [0.4-1.2]	0.9 [0.6-1.5]	NO
Pancreatitis	6	0.7 [0.3-1.8]	1.5 [0.6-3.6]	NO
Angioedema	3	0.7 [0.2-2.7]	1.1 [0.3-3.5]	NO
Asthenia	12	0.6 [0.3-1.2]	1.1 [0.6-2.0]	NO
Dizziness	12	0.6 [0.3-1.2]	0.8 [0.4-1.5]	NO
Decreased appetite	4	0.6 [0.2-2.0]	1.3 [0.5-3.7]	NO
Insomnia	4	0.6 [0.2-1.8]	1.0 [0.3-2.7]	NO
Paraesthesia	5	0.6 [0.2-1.8]	0.9 [0.3-2.3]	NO
Neck pain	3	0.6 [0.1-2.2]	1.2 [0.4-4.1]	NO
Chest discomfort	3	0.6 [0.1-2.1]	0.8 [0.2-2.6]	NO
Hyperhidrosis	5	0.5 [0.2-1.5]	1.0 [0.4-2.5]	NO
Cerebrovascular accident	5	0.5 [0.2-1.3]	0.5 [0.2-1.2]	NO
Hypersensitivity	6	0.5 [0.2-1.2]	0.6 [0.2-1.4]	NO
Abdominal pain	9	0.5 [0.2-1.0]	0.8 [0.4-1.6]	NO
Gait inability	3	0.5 [0.1-1.8]	1.7 [0.5-5.6]	NO
Urticaria	4	0.5 [0.1-1.4]	0.5 [0.1-1.4]	NO
Dyspnoea	10	0.4 [0.2-0.9]	0.6 [0.3-1.2]	NO
Atrial fibrillation	3	0.4 [0.1-1.6]	0.7 [0.2-2.4]	NO
Chest pain	6	0.4 [0.1-1.0]	0.7 [0.3-1.7]	NO
Hypertension	3	0.3 [0.1-1.2]	0.5 [0.1-1.7]	NO
Memory impairment	3	0.3 [0.1-1.1]	0.5 [0.1-1.7]	NO
Feeling abnormal	5	0.3 [0.1-1.0]	0.7 [0.3-1.7]	NO

Vomiting	3	0.3 [0.0-1.0]	0.5 [0.1-1.6]	NO
Influenza	8	0.2 [0.1-0.6]	0.3 [0.1-0.6]	NO
Urinary tract infection	4	0.2 [0.0-0.7]	0.8 [0.2-2.1]	NO
Cough	3	0.2 [0.0-0.7]	0.3 [0.1-0.9]	NO
Nasopharyngitis	3	0.2 [0.0-0.6]	0.3 [0.1-0.9]	NO
Hyperphagia	3	-	9.8 [2.5-37.1]	NO
Papule	3	-	8.7 [2.3-32.3]	NO
Injection site haematoma	4	-	4.3 [1.5-12.6]	NO
Fluid retention	3	-	3.9 [1.1-13.2]	NO
Dermatitis allergic	3	-	3.1 [0.9-10.4]	NO
Gout	3	-	3.0 [0.9-10.0]	NO
Injection site urticaria	3	-	2.8 [0.8-9.2]	NO
Skin reaction	3	-	2.6 [0.7-8.5]	NO
Feeling cold	3	-	2.5 [0.7-8.3]	NO
Coronavirus infection	4	-	17.4 [4.9-62.1]	NO
Low density lipoprotein abnormal	12	-	13.2 [6.6-26.7]	NO
Movement disorder	5	-	11.9 [4.1-34.4]	NO
Toothache	4	-	11.6 [3.5-37.9]	NO
Blood uric acid increased	4	-	-	NO

no-norm., no-normalized analysis; norm., normalized analysis; PT, preferred term; R, reported; RG, reference group; ROR, reporting odds ratio; Exp., ADR expectedness based on inclisiran European Medicines Agency SmPC.

Supplementary Material Table S2. The 20 most frequent PTs observed in RG in the non-normalized analysis not reported in inclisiran.

**Non normalized analysis**	
**PT**	**N (%)**
Wrong technique in product usage process	418 (2.8)
Myocardial infarction	361 (2.5)
Product dose omission issue	286 (1.9)
Rhinorrhoea	283 (1.9)
Device difficult to use	266 (1.8)
Accidental exposure to product	244 (1.7)
Pneumonia	206 (1.4)
Oropharyngeal pain	199 (1.4)
Fall	175 (1.2)
Injection site bruising	167 (1.1)
Condition aggravated	154 (1.0)
Diabetes mellitus	144 (1.0)
Injection site haemorrhage	129 (0.9)
Product storage error	122 (0.8)
Unevaluable event	118 (0.8)
Device issue	118 (0.8)
Drug dose omission by device	117 (0.8)
Blood glucose increased	113 (0.8)
Cardiac disorder	108 (0.8)
Hospitalization	108 (0.8)

PT, preferred term;

Supplementary Material Table S3. The 20 most frequent PTs observed in RG in the normalized analysis not reported in inclisiran.

**Normalized analysis**	
**PT**	**N (%)**
Myocardial infarction	57 (3.3)
Pneumonia	37 (2.2)
Oropharyngeal pain	37 (2.2)
Product dose omission issue	29 (1.7)
Rhinorrhoea	27 (1.6)
Amnesia	24 (1.4)
Angina pectoris	23 (1.3)
Cardiac disorder	21 (1.2)
Condition aggravated	19 (1.1)
Chills	19 (1.1)
Product use issue	19 (1.1)
Fall	17 (1.0)
Death	17 (1.0)
Confusional state	17 (1.0)
Wrong technique in product usage process	16 (0.9)
Hospitalization	16 (0.9)
Injection site bruising	13 (0.8)
Sinusitis	13 (0.8)
Dysphonia	13 (0.8)
Abdominal distension	13 (0.8)

PT, preferred term;
